# Racial justice and HIV in the United States: now is the time

**DOI:** 10.1002/jia2.25625

**Published:** 2020-10-19

**Authors:** Bria A Godley, Victor J Schoenbach, Adaora A Adimora

**Affiliations:** ^1^ UNC School of Medicine The University of North Carolina at Chapel Hill Chapel Hill NC USA; ^2^ Department of Epidemiology UNC Gillings School of Global Public Health The University of North Carolina at Chapel Hill Chapel Hill NC USA; ^3^ Department of Medicine UNC School of Medicine The University of North Carolina at Chapel Hill Chapel Hill NC USA

**Keywords:** HIV, racial justice, United States, police reform, criminal justice reform, healthcare reform

Abundant evidence demonstrates the effects of racism in producing and maintaining the nation’s persistent, dramatic racial inequities in rates of HIV infection, cardiovascular disease, cancer and death [1]. Racism has defined the lives of Black people in America for centuries. In recent years, however, instant dissemination of cellphone videos has put the almost routine extrajudicial executions of Black Americans on vivid display. This past spring, the video of George Floyd’s murder by police galvanized the nation into beginning to recognize the impact of racism on Black Americans. Protests for racial justice occurred in more than 2500 US cities and 40% of the nation’s counties, with participation of an estimated 15 to 26 million people, making these recent protests possibly the largest in US history [2].

Significant changes have already ensued. For example, the Minneapolis City Council promised to dismantle its police department and create a new public safety system [3]. New York State repealed a law that kept police disciplinary records secret [4]. Mississippi removed its state flag that bore the Confederate emblem [5].

## NOW IS THE TIME

This moment can catalyse more profound, long‐overdue reforms in two areas critical for the health and welfare of Black and Brown Americans. These areas are particularly important with regard to HIV. An estimated 14% of people with HIV traverse US correctional or detention facilities each year [6]. The *criminal justice system*, especially the disproportionate incarceration of Blacks, affects individual and community health, including HIV risk, through numerous diverse pathways, such as obstructing educational and career opportunity, thereby increasing unemployment and poverty; disrupting stable monogamous relationships, thereby fostering sexual network patterns that promote HIV transmission; and disenfranchising a significant proportion of the Black population, thereby reducing their influence on public policies [7, 8, 9, 10]. *Health care access* affects virtually all aspects of prevention and care for HIV and other conditions. We suggest four targets for interventions that could start immediately and substantially reduce HIV risk and improve personal and public health.

## 1 REFORM POLICING

Systemic reform of policing is critical; several key areas should be targeted immediately. First, relatively few reliable data document the numbers of people killed by law enforcement, and police have had a vested interest in obscuring the scope of these murders. However, as Krieger notes, police killings are public health data and should be counted as such [11]. Second, individual police and the law enforcement agencies they represent must be held accountable for police actions and misconduct. Third, state and local police have become increasingly militarized – generally in the absence of public oversight, with the use of paramilitary teams and weapons. The American Civil Liberties Union reports that neighborhoods in some cities are considered “war zones” whose residents are treated “like wartime enemies” [12]. This militarization, whose tragic effects have been largely visited on communities of colour, must be halted [12]. Fourth, a substantial proportion of adverse civilian interactions with police are related to social and psychiatric problems. Funds should be reallocated from police militarization to housing, mental health treatment, greater use of social and mental health workers, and an expanded social safety net.

## 2 IMPLEMENT POLICIES THAT PROMOTE DECARCERATION

The United States incarcerates more people and has higher incarceration rates per capita than any other country, with 2.2 million people (roughly 0.7% of the population) in jail or prison [13]. The US prison population increased 500% from the 1980s through the present – despite decreases in the national crime rate – and evidence that mass incarceration is not an effective strategy for achieving public safety [13, 14]. Communities of colour disproportionately bear the brunt of incarceration and its effects. The lifetime risk of incarceration for a man born in 2001 is 1 in 17 for Whites, compared to 1 in 6 for Latinx and 1 in 3 for Blacks [13]. The United States maintains its high incarceration rates through a variety of means, typically involving laws and policies that require, for example: long sentences (including increases in the number of life sentences), mandatory minimum sentences, and incarceration for low‐level drug and property crimes and failure to pay court fees and fines. Mass incarceration is expensive, and in recent years stakeholders from diverse parts of the political spectrum have come to recognize incarceration’s drain on government budgets, especially its depletion of funds that could be used for education and other critical services. Some states have begun to decrease their prison populations through changes in policy, such as elimination of some mandatory minimum sentences, specialty courts and other alternatives to incarceration, and reductions in reincarceration for violation of technical terms of parole [14, 15]. While incarceration in these states has decreased somewhat, the impact of these changes on the nation’s overall incarceration rate has been modest. Ongoing efforts are needed, with particular attention to decreasing persistent racial disparities [14].

## 3 ELIMINATE COLLATERAL CONSEQUENCES OF INCARCERATION

Collateral consequences, including the legal disabilities resulting from a conviction, are substantial and far‐reaching. For example in most states people with a felony conviction lose the right to vote – which, given the disproportionate incarceration of Black people, affects Black representation in the electorate and may have determined the outcome in Senate and presidential elections [16]. Criminal convictions can also deny people access to governmental programmes, such as access to student loans, federally assisted housing and food assistance, including Temporary assistance for Needy Families (TANF) and Supplemental Nutrition Assistance Program (SNAP) [17]. These and other laws and policies that thwart successful reentry to the community can and should be changed, as they increase poverty and likelihood of reincarceration and exacerbate health inequities, including for HIV.

## 4 PROVIDE ACCESS TO HIGH‐QUALITY HEALTHCARE FOR ALL

Health insurance, typically provided by employers, is essential for accessing health care in the United States. The Affordable Care Act (ACA), the comprehensive healthcare reform law whose primary goal was to make affordable healthcare insurance available to more people, was arguably the single biggest structural intervention in the United States for prevention and care for HIV infection and other conditions. Between the ACA’s enactment in 2010 and 2018, the ethnic groups most likely to be uninsured and to have the worst health indicators had their coverage rates increase from about 57% to 73% (among Latinos) and about 72% to 85% (among Blacks) [18]. However, the ACA has been under constant political assault since its passage. The Republican Party has consistently sought to undermine the ACA, and in 2018 the proportion of Americans with health insurance fell for the first time since the ACA was implemented, despite the strong economy at that time [19]. Since the beginning of the Coronavirus pandemic, more than 36 million Americans lost their jobs, including 11% of White, 20% of Latinx, and 24% of Black workers [20, 21]. As a result, an estimated 27 million people lost health insurance [22]. The percent of laid‐off workers who lost insurance in the 13 states that did not expand Medicaid under the ACA (43%) was nearly double the percentage in the 37 states that expanded Medicaid (23%) [23, 24]. Clearly, Latinos and Black Americans – and in fact all Americans – need comprehensive health insurance immediately.

The interventions highlighted above will not reverse centuries of oppression, but they are necessary, can be undertaken immediately, and will weaken fundamental drivers of the HIV epidemic in the United States as well as improve life for all Americans. Effective public health requires effective government, and in the words of Congressman John Lewis, “a democracy cannot thrive where power remains unchecked and justice is reserved for a select few” [25].

## COMPETING INTERESTS

AAA has received consulting fees from Merck, Viiv and Gilead. Gilead has provided her institution with funding for her research. BAG and VJS have no conflicts of interests to declare.

## AUTHORS’ CONTRIBUTIONS

BAG and AAA conducted the research for this article. All authors contributed to writing and revisions.
